# Development and Validation of the Communities Geriatric Mild Cognitive Impairment Risk Calculator (CGMCI-Risk)

**DOI:** 10.3390/healthcare12202015

**Published:** 2024-10-10

**Authors:** Jiangwei Chen, Qing Fang, Kehua Yang, Jiayu Pan, Lanlan Zhou, Qunli Xu, Yuedi Shen

**Affiliations:** 1 School of Nursing, Hangzhou Normal University, Hangzhou 311121, China; 2022112024028@stu.hznu.edu.cn (J.C.); 2022112024043@stu.hznu.edu.cn (Q.F.); 2 Nursing Department, Sir Run Run Shaw Hospital, Zhejiang University School of Medicine, Hangzhou 310016, China; yangkh@srrsh.com; 3 School of Clinical Medicine, Hangzhou Normal University, Hangzhou 311121, China; 2022112028012@stu.hznu.edu.cn; 4 Department of Neurology, Sir Run Run Shaw Hospital, Zhejiang University School of Medicine, Hangzhou 310016, China; 3204026@zju.edu.cn

**Keywords:** mild cognitive impairment, cognitive disorder, cognitive function, community health, healthcare, prediction model, risk model

## Abstract

**Objectives**: The aim was to develop and validate the Communities Geriatric Mild Cognitive Impairment Risk Calculator (CGMCI-Risk), aiding community healthcare workers in the early identification of individuals at high risk of mild cognitive impairment (MCI). **Methods**: Based on nationally representative community survey data, backward stepwise regression was employed to screen the variables, and logistic regression was utilized to construct the CGMCI-Risk. Internal validation was conducted using bootstrap resampling, while external validation was performed using temporal validation. The area under the receiver operating characteristic curve (AUROC), calibration curve, and decision curve analysis (DCA) were employed to evaluate the CGMCI-Risk in terms of discrimination, calibration, and net benefit, respectively. **Results**: The CGMCI-Risk model included variables such as age, educational level, sex, exercise, garden work, TV watching or radio listening, Instrumental Activity of Daily Living (IADL), hearing, and masticatory function. The AUROC was 0.781 (95% CI = 0.766 to 0.796). The calibration curve showed strong agreement, and the DCA suggested substantial clinical utility. In external validation, the CGMCI-Risk model maintained a similar performance with an AUROC of 0.782 (95% CI = 0.763 to 0.801). **Conclusions**: CGMCI-Risk is an effective tool for assessing cognitive function risk within the community. It uses readily predictor variables, allowing community healthcare workers to identify the risk of MCI in older adults over a three-year span.

## 1. Introduction

Mild cognitive impairment (MCI) is an intermediate stage between normal aging and dementia, often considered a clinical state that precedes Alzheimer’s disease (AD) [1,2]. The global prevalence of MCI among community-dwelling adults aged 50 and older was over 15% [3]. In China, the prevalence of MCI among individuals aged 60 and above is 15.5%, with the number of affected reaching 38.77 million [4]. It is estimated that over 50% of individuals diagnosed with MCI will develop dementia within five years, with only a small percentage maintaining stable cognitive function [5]. In the absence of timely diagnosis, patients may exhibit impairments in two or more cognitive domains, such as memory, language, executive function, perceptual speed, and visuospatial abilities [6,7,8]. These impairments can severely affect the patient’s independence in daily life and place a significant burden on caregivers and families, ultimately increasing the societal burden [9]. Existing MCI detection methods often rely on clinical assessment and neuropsychological tests. Although these methods can provide some diagnostic basis, it is difficult to accurately identify subtle cognitive changes in the early stage of the disease. This leads to the failure of many potential MCI patients to receive timely identification and intervention. Therefore, it is crucial to identify individuals at high risk for MCI as early as possible.

Previous studies have illuminated a multitude of risk factors that significantly impact cognitive function in older adults, spanning demographic, physical health, lifestyle, biological, and genetic domains. Demographic characteristics, including age, sex, educational level, and marital status, have consistently emerged as influential predictors of cognitive impairment [10,11]. These factors set the foundational context for understanding individual variability in cognitive trajectories. Physical health conditions have been robustly linked to cognitive decline. Research has documented associations between reduced cognitive function and limitations in both basic activities of daily living (BADL) [12] and instrumental activities of daily living (IADL) [13], highlighting the importance of functional status. Furthermore, obesity, as measured by body mass index (BMI) [14], hypertension [15], and sensory impairments such as vision [16] and hearing loss [17] have been identified as risk factors. Chronic diseases, notably diabetes and stroke [18], exacerbate this risk landscape, emphasizing the need for holistic health management. Lifestyle factors play a pivotal role in modulating cognitive function. Regular exercise [19] and healthy dietary habits [20] have been shown to positively influence cognitive outcomes, while smoking and excessive alcohol consumption [21] exert detrimental effects. These modifiable behaviors offer promising avenues for intervention to promote cognitive health. At the biological level, sex hormones [22] and hemoglobin levels [23] have been implicated in cognitive dysfunction, suggesting intricate physiological mechanisms underlying cognitive decline. Moreover, the relationship between mental health and cognition is underscored by the strong correlation between depressive states and cognitive decline reported by Ferri et al. [24]. Genetic factors also contribute to cognitive risk. Notably, Gui et al. [25] have identified the APOEε4 allele as a clear risk factor for mild cognitive impairment (MCI), emphasizing the role of individual genetics in disease susceptibility. Given the myriad of factors influencing cognitive function in the elderly, developing a prediction model for MCI becomes paramount. Predictive models play an important role in primary prevention, and their main purpose is to detect and treat diseases in time to avoid disease progression and deterioration [26].

Previous studies have proposed a variety of MCI prediction models, but these models have certain limitations and challenges. Huang et al. [27] developed an MCI prediction model using data from 478 community-dwelling middle-aged and older adults (≥45 years old). The predictors included age, sex, educational level, place of residence, and reading, with an area under the receiver operating characteristic curve (AUROC) of 0.870. This model lacks external validation, and its predictive effect may be biased. Ma et al. [28] developed an MCI risk prediction model for older adults (≥60 years old) using public datasets. This risk prediction model used different MCI assessment methods during the development and validation phases, which may impact the stability and accuracy of the model. Additionally, MCI prediction models have been developed for other specific patient populations, including those with hypertension [29], diabetes [30], and stroke [31]. While prediction tools for these populations are available, variations in study populations and study designs have resulted in a diversity of model variables. MCI prediction models have also been developed using data from various data sources, including neuro biomarkers like A-β amyloid [32] and tau protein [33], neuroimaging variables such as brain microstructure [34], and genetics such as mitochondria-related genes [35]. The predictive accuracy of these models has significantly improved; however, implementing these biomarker tests in community settings remains challenging. Consequently, the objective of this study was to develop and validate a risk prediction model, known as CGMCI-Risk, for MCI in community-dwelling older adults. CGMCI-Risk can be used by community healthcare workers to predict MCI risk in community-dwelling older adults as well as time-validated models (a shortcoming of many models). Most current prediction models are presented as a nomogram, which can make them difficult and inconvenient to use. The CGMCI-Risk automates the calculations and provides fast predictions compared to manual calculations or the use of traditional tools. This model aims to help community healthcare professionals identify high-risk MCI groups, thereby facilitating the optimization of prevention and intervention strategies.

## 2. Materials and Methods

### 2.1. Dataset

The data were obtained from the Chinese Longitudinal Healthy Longevity Survey (CLHLS). The CLHLS is one of the largest national longitudinal studies examining the health status of older adults in China. It includes eight surveys conducted in 23 provinces, municipalities, and autonomous regions between 1998 and 2018. The sampling area covered approximately 85% of China’s total population [36]. The CLHLS dataset includes basic information, health assessments, personality traits, lifestyle factors, personal backgrounds, and indicators of physical health. All eligible participants who consented signed an informed consent form. For older adults unable to sign the form themselves, consent was provided by a family member on their behalf.

### 2.2. Design and Participants

**Figure 1:**
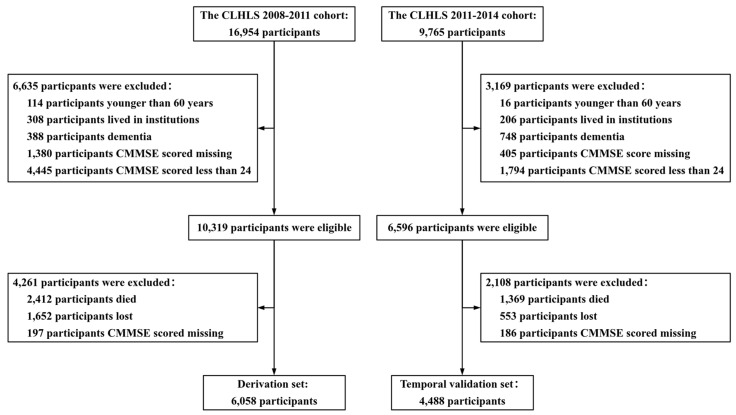
The flowchart of the participants’ selection process. CMMSE, Chinese Mini-Mental State Examination.

In this study, the CLHLS cohort data from 2008 to 2011 were selected as the derivation set, and the CLHLS cohort data from 2011 to 2014 were used as the temporal validation set for the model. The study population included community-dwelling older adults aged 60 years and older with healthy cognition. Individuals who were institutionalized or who self-reported or were diagnosed with dementia were excluded. Figure 1 presents a flowchart of sample selection in the present study. This study was conducted in accordance with the TRIPOD reporting specifications [37].

### 2.3. Assessment of MCI

Cognitive functioning was assessed using the Chinese Mini-Mental State Examination (CMMSE), which consists of 24 items assessing general cognitive ability, reactivity, attention and calculation, recall, and language comprehension and coordination. Each correct response earns one point, while incorrect responses receive zero points. Question 6, “Count the number of food groups in one minute”, has a maximum score of 7, making the total score range from 0 to 30. Based on previous research [38,39], the CMMSE score below 24 was considered as MCI.

### 2.4. Definition of Candidate Variables

In accordance with previous research on cognitive dysfunction in older adults [10,40,41], 41 candidate variables were selected from the CLHLS survey data for analysis in this study. The variables were grouped into four categories. (1) Demographic characteristics: These include age, educational level, sex, ethnic group, place of residence, marital status, and cohabitant status. (2) Health status and lifestyle: These include BMI, medical expenses, baseline CMMSE score, sleep duration, BADL, IADL, physical performance test (PPT), chronic diseases, masticatory function, vision, hearing, frequency of intake of fruits, fresh vegetables, animal protein, plant protein and tea, house work, field work, garden work, raise domestic animals or pets, read newspapers or books, TV watching or radio listening, playing cards or mahjong, social activities, smoke, alcohol use, and exercise. (3) Mental health: It includes resilience score, life satisfaction, health satisfaction, sleep satisfaction, and financial satisfaction. (4) Community and family support: This includes child support and community services. See Supplementary Materials Table S1 for information on candidate variables.

### 2.5. Sample Size

The study was designed to achieve an AUROC of at least 0.7 [42]. A total of 5470 participants and 821 outcome events were calculated using the R package “pmsampsize” (version 1.1.3), in accordance with the methodology outlined by Riley et al. [43].

### 2.6. Missing Value

Values marked as “missing”, “unclear”, and “unanswerable” were considered as missing data. Individuals with more than 5% of their variables missing were excluded from the analysis. Missing values were imputed using the nearest neighbor (KNN) method, which involves estimating the value of a missing data point based on the mean (for numeric variables) or the most common (for categorical variables) value observed in the K participants who are most similar to the participant with the missing value [44]. In this study, the value of K was set to 5.

### 2.7. Statistical Analysis

The study data were statistically analyzed using R (version 4.3.2). The numeric variables were analyzed according to the distribution characteristics of the variables. Variables with approximately normal distributions were described using means and standard deviations. Comparisons between groups were performed using the independent samples *t*-test. Numeric variables with severely skewed distributions were described using medians and interquartile ranges (IQR). Comparisons between groups were performed using non-parametric tests. Categorical variables were described as frequencies and percentages. Comparisons between groups were performed using the chi-square test. All tests were performed with two-sided *p* < 0.05 as statistically significant differences. The R packages used in this study include “pmsampsize” (version 1.1.3), “VIM” (version 6.2.2),”tableone” (version 0.13.2), “MASS” (version 7.3-60), “rms” (version 6.7-1), “pROC” (version 1.18.5), “rmda” (version 1.6), “caret” (version 6.0-94), and “nomogramEx” (version 3.0).

### 2.8. Model Development and Validation

A backward stepwise regression analysis was employed to screen the variables in accordance with the Akaike Information Criterion (AIC) minimization principle. The screened predictor variables were then included in the first multivariate logistic regression analysis, and subsequently, significant predictor variables were included in the second logistic regression analysis until all predictor variables in the model were shown to be significant. The CGMCI-Risk model was developed using logistic regression. Scores for each variable were extracted using the R package “nomogramEx” (version 3.0), which contributed to the formation of the CGMCI-Risk model.

The AUROC [45], calibration curve [46], and DCA [47] were employed to evaluate the discriminatory power, calibration, and clinical utility of CGMCI-Risk, respectively. The AUROC ranged from 0 to 1, with a value of 1 indicating 100% correct predictions [45]. The Hosmer–Lemeshow (H-L) goodness-of-fit (GOF) test was employed to construct calibration plots, which were utilized to assess the degree of agreement between the predicted risk and the actual status of MCI. In a calibration plot, the x-axis represents the predicted probability, the y-axis represents the observed probability, and the diagonal line represents a perfect prediction [48]. DCA calculates the net benefit through a series of risk-probability thresholds and analyzes the value and consequences of the decision under consideration [49]. The robustness of the model was evaluated through the application of bootstrap resampling and temporal validation. The accuracy, sensitivity, specificity, negative predictive value (NPV), and positive predictive value (PPV) of CGMCI-Risk were subsequently reported.

## 3. Results

### 3.1. Participants

Of the 6058 participants in the derivation set (median (IQR) age was 79 (71, 87) years), 933 (16.3%) developed MCI within 3 years, 2963 (48.9%) were female and 3095 (51.1%) were male. Of the 4448 participants in the temporal validation set (median (IQR) age was 79 (73, 87) years), 635 (14.1%) developed MCI within 3 years, 2205 (49.1%) were female and 2283 (50.9%) were male. More cohort characteristics of the derivation set and temporal validation set are presented in Supplementary Materials Table S2.

### 3.2. CGMCI-Risk Development and Validation

The set of variables with a minimum AIC value of 4548 was screened by backward stepwise regression analysis. The first multivariate logistic regression analysis was performed using the screened variables, the statistically insignificant variables were removed, and the multivariate logistic regression model was reconstructed using the remaining significant variables. The eventually multivariate logistic regression model included age, educational level, sex, exercise, garden work, TV watching or radio listening, IADL, hearing, and masticatory function. The results are shown in Supplementary Materials Table S3. Based on this model, the CGMCI-Risk tool was developed to calculate disease scores, with personalized results obtained by inputting the appropriate values or options. The corresponding scores for each parameter are presented in Supplementary Materials Table S4. The equation that describes the relationship between the predicted risk value and the sum of the parameter scores is as follows:

Risk = 0.852405178 − 4.17 × 10^−7^ × Total Points^3^ + 0.000194207 × Total Points^2^ − 0.022360467 × Total Points (1)

We provide this CGMCI-Risk model with a user-friendly web platform that enables the estimation of normative deviation scores from any sample with minimal technical and computing requirements: http://g152335m31.imdo.co/Communities Geriatric Mild Cognitive Impairment Risk Calculator (accessed on 4 October 2024). The community medical staff can utilize this link to input the corresponding predictor variables and obtain an individualized three-year risk assessment value for MCI in the elderly residing within the community. Based on the evaluation results, targeted health guidance can be provided by the community medical staff to reduce the incidence of MCI.

**Figure 2:**
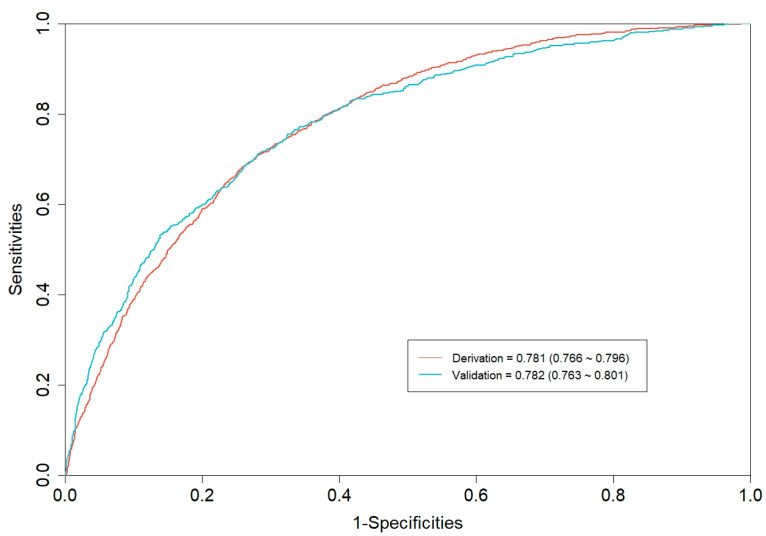
The AUROC for derivation set and temporal validation set of CGMCI-Risk.

**Figure 3:**
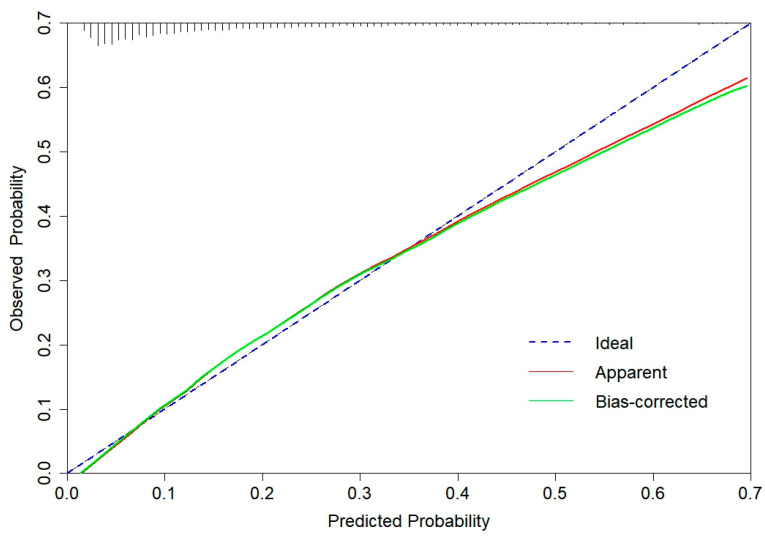
The calibration curve for derivation set of CGMCI-Risk.

**Figure 4:**
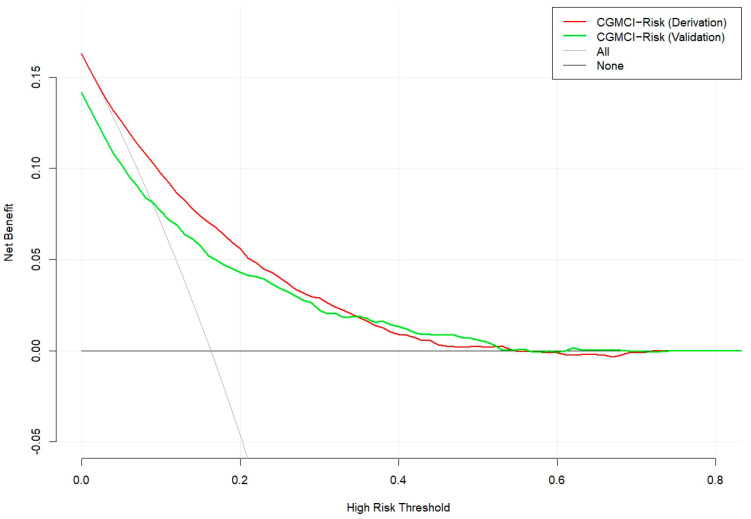
The DCA for derivation set and temporal validation set of CGMCI-Risk.

The CGMCI-Risk model demonstrated robust discriminatory power in identifying MCI risk in community-dwelling older adults, with an AUROC of 0.781 (Figure 2). This indicates that in a scenario where one randomly selected high-risk patient and one low-risk patient are considered, the model has approximately a 78.1% probability of correctly identifying the high-risk patient. The accuracy, sensitivity, specificity, NPV, and PPV of CGMCI-Risk in the derivation set were 0.717, 0.710, 0.719, 0.927, and 0.330, respectively. The study employed the H-L GOF test to assess the consistency between the model’s predicted probability and the actual probability (*p* = 0.146). Calibration plots were also constructed to facilitate this assessment. The results demonstrated a robust correlation between the actual and predicted probabilities, with a mean absolute error (MAE) of 0.011 and a mean square error (MSE) of 0.00023 (Figure 3). It indicates that the prediction result of CGMCI-Risk is very close to the actual result, and the prediction accuracy is high. The DCA illustrated that CGMCI-Risk offers a greater net benefit compared to full treatment or no treatment when the risk threshold ranges from 4% to 57% (Figure 4).

**Figure 5:**
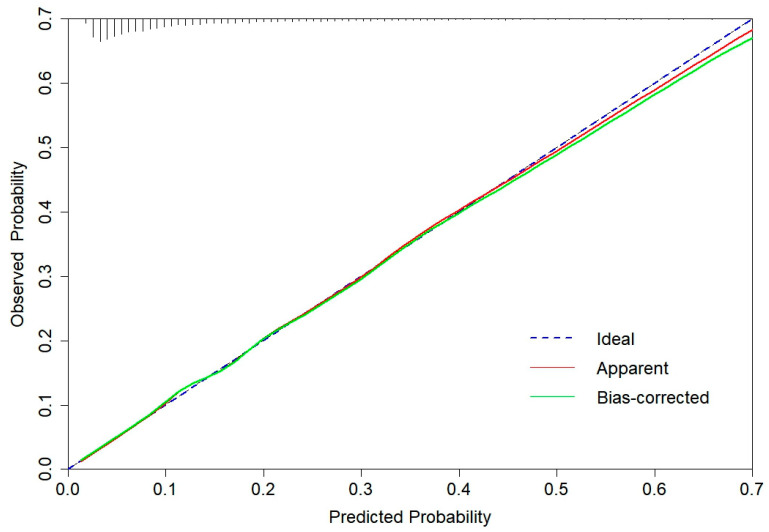
The calibration curve for the temporal validation set of CGMCI-Risk.

Internal validation employed bootstrap resampling 1000 times with AUROC, sensitivity, and specificity of 0.776, 0.977, and 0.125, respectively. The temporal validation set was conducted using data from the CLHLS cohort from 2011 to 2014. The AUROC, sensitivity, and specificity were 0.782, 0.765, and 0.666, respectively (Figure 2). The calibration curve and DCA are similar to the derivation set indicating that the CGMCI-Risk is an effective tool for identifying older adults at risk for MCI in the community (Figure 4 and Figure 5), thereby providing a foundation for early cognitive intervention by community healthcare workers.

## 4. Discussion

### 4.1. Main Findings

In this study, CGMCI-Risk was developed based on a dataset comprising 6058 samples, and its external validity was subsequently temporal validated with an additional 4488 samples. The AUROC values demonstrated consistent performance, with a mean of approximately 0.8, indicating a good discriminative ability. The results of AUROC difference detection between the derived set and the temporal validation set show that the slight difference between the two may be just random fluctuation (*p* = 0.988). The calibration curve demonstrated excellent consistency, and DCA validated its utility, establishing a robust tool for MCI risk assessment in community-dwelling older adults. The CGMCI-Risk incorporates age, educational level, sex, exercise, garden work, TV watching or radio listening, IADL, hearing, and masticatory function.

Age and sex are significant non-intervention factors in cognitive impairment. The prevalence of MCI increases with age. As a consequence of the aging process, the volume of the cerebral cortex and hippocampus diminishes [50]. This results in a blockage of information delivery, which in turn impairs cognitive function [50]. Yesavage et al. [51] modeled the prevalence and incidence of AD and MCI. The primary findings of the model include that the conversion rate from a normal cognitive state to MCI increased from 1% per year at age 60 to 11% at age 85. This suggests that age is a significant risk factor for the development of MCI. A meta-analysis of the association between sex and MCI revealed that women are a risk factor for MCI [52]. The role of estrogen in neurogenesis in the hippocampus is significant, and fluctuations in its levels may be associated with an increased risk of MCI in females [53]. Furthermore, women are more prone to the formation of ApoEε4-associated neurogenic fiber tangles, which may contribute to an elevated risk of cognitive impairment [54]. A review of the literature reveals a correlation between educational attainment and a number of factors related to cognitive functioning, including the thickness of the cerebral cortex, gray matter volume, and neural network connectivity [55]. Individuals with higher levels of education tend to demonstrate superior cognitive functioning [56], whereas illiteracy or lower educational attainment represents a substantial risk factor for MCI [57].

There is a strong correlation between IADL and cognitive function. As IADL declines, older adults may also experience a decline in cognitive abilities [13]. This association may be attributed to the fact that sustained stimulation of cerebral function through IADL preserves the activity and plasticity of the brain’s neural networks, thereby assisting in the mitigation of cognitive decline [58]. A reduction in IADL may also result in a decline in socialization among older adults, which may further impact their cognitive function [59]. Hearing impairment represents a significant risk factor for the onset of MCI. A study investigating the impact of hearing on cognitive function demonstrated that individuals with normal hearing exhibited superior performance on cognitive assessments [60]. This may be attributed to the fact that hearing impairment can result in alterations to brain structure and function [17]. Examples of these changes include a decline in brain signals, degeneration of the auditory cortex, loss of neurons and neuron branches, and a reduction in overall brain volume [61]. Such alterations may impact the brain’s capacity to process and perceive sound, potentially contributing to cognitive decline. Tooth loss can result in difficulty chewing, which may affect nutrient absorption and cognitive function in the brain [62]. Momose et al. [63] and Onozuka et al. [64] have demonstrated increased hemodynamic responses in the prefrontal cortex and hippocampus during chewing, which plays a crucial role in cognitive function. Research has indicated a correlation between tooth loss, chewing difficulties, and cognitive decline [65], while effective mastication has been shown to have a beneficial impact on the prevention of MCI [66].

Regular exercise has been demonstrated to exert a beneficial influence on the brain [67]. A research study demonstrated that sustained exercise can delay the onset of cognitive impairment in older adults [68]. An intervention study by Kim and colleagues also confirmed that exercise may improve cognitive function in older adults aged 65 and above [19]. Regular exercise has been demonstrated to facilitate the formation of neural connections between regions of the brain that are essential for optimal cognitive function [69]. Furthermore, it facilitates the release of brain-derived neurotrophic factor (BDNF) in the brain, which is instrumental in promoting neuronal growth, connectivity, and maintenance [70,71]. It is hypothesized that gardening may confer benefits with respect to cognitive function in older adults. Findings from a four-year longitudinal study indicate that gardening may be a significant factor in the reversal of MCI in older adults [72]. In addition to providing enriching stimulation [73], gardening has been shown to result in significantly higher levels of BDNF, which can lead to improvements in both physical and cognitive functioning [74]. Furthermore, the role of passive activities such as watching television or listening to the radio in cognitive impairment has been demonstrated. Lin et al. [75] and Major et al. [76] have shown that these activities can significantly improve cognitive performance in older adults. However, Jung et al. [77] posited that television viewing may be associated with an increased risk of cognitive impairment in later life. This may be attributed to the fact that prolonged television viewing is frequently linked to sedentary behavior, which can result in inadequate physical activity or reduced time spent gardening. Consequently, watching television or listening to the radio may become a risk factor [76].

### 4.2. Significance and Application Prospects

Currently, more than 55 million individuals worldwide are affected by dementia, with AD representing approximately 60% to 70% of dementia cases [78]. MCI progresses to AD at a rate of 10% to 15% per year, whereas the rate of transition to AD in normal older adults is only 1% to 2% per year [79]. Although current clinical interventions may not be capable of curing these diseases, timely recognition and diagnosis are essential for improving patient prognosis and reducing the burden on caregivers [80]. According to the 2019–2030 Healthy China Action Plan of the Chinese government, effective measures can be taken to prevent and slow down the occurrence of dementia and reduce the burden on families and society [81]. MCI patients are the key population for the primary prevention of dementia. If CGMCI-Risk is integrated into the existing community healthcare information system, it can be easily accessed and used by the community. This may involve software development, interface docking, and data exchange.

CGMCI-Risk enhances accessibility and feasibility of assessment and optimizes healthcare worker engagement. It is particularly suited to community settings, providing community healthcare workers with a foundational resource for conducting early cognitive interventions. The CGMCI-Risk informs the allocation of healthcare resources based on a patient’s risk score, potentially leading to enhanced resource accessibility for high-risk patients while comparatively neglecting low-risk patients. This “risk-oriented” approach to resource allocation may give rise to equity concerns. Therefore, besides considering the risk score as a crucial factor, it is imperative to comprehensively evaluate the multi-dimensional factors of patients, including but not limited to their socioeconomic background, personal health needs, and preferences, in order to ensure comprehensive and equitable resource allocation. Conversely, older adults identified as high risk by CGMCI-Risk may encounter issues related to stigmatization and be labeled as “high risk” or “susceptible to disease”. To avoid such labeling, it is essential to deepen public understanding of CGMCI-Risk and clarify its role as an auxiliary decision-making tool rather than an absolute standard.

### 4.3. Limitations

The CGMCI-Risk model was developed to identify high-risk groups for MCI in community-dwelling older adults. Although laboratory parameters, imaging features, biomarkers, and genetic indicators have significant potential for MCI prediction, they were excluded from this study due to logistical and operational feasibility in a real-world community setting. In addition, the three-year interval for MCI assessment may introduce bias and fail to capture subtle disease changes. The elevated mortality rate among older adults may also lead to increased data loss and impact assessment precision. The CGMCI-Risk model was developed based on Chinese community-dwelling older adults and, while promising for use in community settings, requires further validation for its applicability in different care facilities and cultural backgrounds.

## 5. Conclusions

The CGMCI-Risk model incorporates factors such as age, educational level, sex, exercise, garden work, TV watching or radio listening, IADL, hearing, and masticatory function. This tool can be used in community settings to help healthcare providers identify older adults in the community at elevated risk for MCI within three years. The role of CGMCI-Risk in facilitating early identification and intervention of MCI among elderly individuals in the Chinese community is crucial. Further validation is required to assess the predictive efficacy of CGMCI-Risk for MCI in elderly populations outside China. Moreover, as healthcare practices advance, longitudinal studies should be conducted to ensure the continued reliability of CGMCI-Risk as a tool. In future research, self-reported data could be cross-validated with objective measurements such as medical records or wearable devices whenever feasible.

## Data Availability

The data were obtained from the CLHLS. Links to public datasets analyzed during the study: https://doi.org/10.18170/DVN/WBO7LK (accessed on 4 October 2024). All scripts for this study can be found on GitHub via the following link: https://github.com/ChenJiangwei2024/CGMCI-Risk (accessed on 4 October 2024).

## Supplementary Materials

The following supporting information can be downloaded at https://www.mdpi.com/article/10.3390/healthcare12202015/s1, Table S1: Definition of candidate variables; Table S2: The cohort characteristics of the derivation set and temporal validation set; Table S3: Multivariate logistic regression analysis of variables associated; Table S4: Points of CGMCI-Risk parameters.
