# Supplementary material for: Development and Validation of the Communities Geriatric Mild Cognitive Impairment Risk Calculator (CGMCI-Risk)
Source: Healthcare (Basel). 2024 Oct 10;12(20):2015. doi: 10.3390/healthcare12202015 (PMC11506964; doi:10.3390/healthcare12202015)
Supplement: Supplementary file 1 [file healthcare-12-02015-s001.zip › healthcare-3187552-supplementary.pdf]

Development and Validation of the Communities Geriatric Mild Cognitive Impairment Risk Calculator (CGMCI-Risk)

Supplementary Materials

Table S1: Definition of candidate variables.

Table S2: The cohort characteristics of the derivation set and temporal validation set.

Table S3: Multivariate logistic regression analysis of variables associated.

Table S4: Points of CGMCI-Risk parameters.

Table S1: Definition of candidate variables.

Candidate variables	Coding	Measure explanation
Demographic characteristics		
Age		Age in chronological years.
Sex	1 = Male;<br>2 = Female	Self-report
Educational level	1= Illiteracy;<br>2 = Primary school;<br>3 = Middle school or above	Self-report
Ethnic group	1 = Han;<br>2 = Non-han	Self-report
Category of residence	1 = City;<br>2 = Town;<br>3 = Rural	Self-report
Marital status	1 = Cohabitation;<br>2 = Single	"Single" include separated, divorced, widowed and never married.
Cohabitant status	1 = With family;<br>2 = Solitude	Self-report
Health status and lifestyle		
BMI	1 = Normal;<br>2 = Abnormal	18.5kg/m^2 ≤ BMI < 24kg/m^2 was defined as "Normal"; BMI ＜ 18.5kg/m^2 or BMI ≥ 24kg/m^2 was defined as "Abnormal".
Medical expenses		Outpatient and inpatient expenses last year.
Baseline CMMSE score	0 = Non-MCI<br>1 = MCI	Cognitive functioning was assessed using the Chinese Mini-Mental State Examination (CMMSE), which consists of 24 items assessing general cognitive ability, reactivity, attention and calculation, recall, and language comprehension and coordination. Each correct response earns one point, while incorrect responses receive zero points. Question 6, "Count the number of food groups in one minute," has a maximum score of seven, making the total score range from 0 to 30. The CMMSE score below 24 was considered as MCI.
Sleep duration		Self-report
Chronic diseases		Hypertension, diabetes, heart disease, stroke, cerebrovascular disease, and respiratory disease, meeting one definition as "Yes".
BADL	1 = Intact;<br>2 = Impaired	Eating, bathing, dressing, toileting, indoor activities, and bowel and bladder control, did not require assistance was defined as "Intact".
IADL		Alone around the neighborhood, shopping, cooking, laundry, alone continuous walking 1 kilometer, raised about 5 kilograms, squat down stand up 3 consecutive, alone in a public transport. No difficulty is defined as "Intact".
PPT		Rising from a chair, picking up a book on the floor, and turning around on their own, did not require assistance and was defined as "Intact".
Masticatory function		Teeth ≥20 or with dentures were defined as "Intact".
Vision		No need for eyeglasses is defined as "Intact".

Candidate variables	Coding	Measure explanation
Hearing		No need for hearing aids is defined as "Intact".
Eat fresh fruits	1 = Usually;<br>2 = Sometimes;<br>3 = Never	Almost every day is defined as "usually"; Rarely or never is defined as "Never"; The remaining options were defined as "Sometimes". Animal protein includes fish, meat, eggs, and dairy products. Plant protein includes bean products and nuts. House work such as cooking, taking care of children, etc.
Eat fresh vegetables		
Eat animal protein		
Eat plant protein		
Drink tea		
House work		
Field work		
Garden work		
Raise domestic animals or pets		
Read newspapers or books		
TV watching or radio listening		
Play cards or mahjong		
Social activities		
Smoke	1 = Yes; 2 = No	Self-report
Alcohol use	1 = Yes; 2 = No	Self-report
Exercise	1 = Yes; 2 = No	It refers to purposeful fitness activities, such as walking, playing ball, running, qigong, etc.
Mental health		
Resilience score		The resilience score was assessed with five questions. Problems included being able to see things positively, feeling scared or anxious, feeling lonely, being able to make personal decisions and feeling useless as you age. Each question is scored on a scale of 1 (always) to 5 (never), and questions 1 and 4 were reverse coded in this study, with total scores ranging from 5 to 25, with higher scores indicating better psychological resilience.
Life satisfaction	1 = Good;<br>2 = So so;<br>3 = Bad	Self-report
Health satisfaction		
Sleep satisfaction		
Financial satisfaction	1 = Rich;<br>2 = So so;<br>3 = Poor	Self-report
Community and family support		
Child support		Money received from children in the last year.
Community services	1 = Yes;<br>2 = No	Community services included daily care, medical treatment and medicine delivery, spiritual comfort, chatting and recreation, daily shopping, organizing social and recreational activities, upholding rights, providing health knowledge, and dealing with family and neighborhood disputes, meeting one definition as "Yes".

Abbreviations: BMI, Body Mass Index; CMMSE, Chinese Mini-Mental State Examination; BADL, Basic Activity of Daily Living; IADL, Instrumental Activity of Daily Living; PPT, Physical Performance Test.

Table S2: The cohort characteristics of the derivation set and temporal validation set.

Variables		Derivation set (n = 6058)				Temporal validation set (n = 4488)			
		Overall n=6058	Non-MCI n=5071	MCI n=987	*p*	Overall n=4488	Non-MCI n=3853	MCI n=635	*p*
Demographic characteristics									
Age		79.00 (71.00, 87.00)	77.00 (70.00, 85.00)	88.00 (81.00, 93.00)	<0.001	79.00 (73.00, 87.00)	78.00 (72.00, 85.00)	89.00 (81.50, 95.00)	<0.001
Sex	Male	3095 (51.1)	2741 (54.1)	354 (35.9)	<0.001	2283 (50.9)	2076 (53.9)	207 (32.6)	<0.001
	Female	2963 (48.9)	2330 (45.9)	633 (64.1)		2205 (49.1)	1777 (46.1)	428 (67.4)	
Educational level	Illiteracy	2961 (48.9)	2248 (44.3)	713 (72.2)	<0.001	2175 (48.5)	1729 (44.9)	446 (70.2)	<0.001
	Primary school	2216 (36.6)	1993 (39.3)	223 (22.6)		1668 (37.2)	1519 (39.4)	149 (23.5)	
	Middle school or above	881 (14.5)	830 (16.4)	51 (5.2)		645 (14.4)	605 (15.7)	40 (6.3)	
Ethnic group	Han	5642 (93.1)	4734 (93.4)	908 (92.0)	0.140	4242 (94.5)	3649 (94.7)	593 (93.4)	0.208
	Non-han	416 (6.9)	337 (6.6)	79 (8.0)		246 (5.5)	204 (5.3)	42 (6.6)	
Category of residence	City	1106 (18.3)	950 (18.7)	156 (15.8)	0.001	688 (15.3)	595 (15.4)	93 (14.6)	0.839
	Town	1243 (20.5)	1069 (21.1)	174 (17.6)		1423 (31.7)	1217 (31.6)	206 (32.4)	
	Rural	3709 (61.2)	3052 (60.2)	657 (66.6)		2377 (53.0)	2041 (53.0)	336 (52.9)	
Marital status	Cohabitation	2995 (49.4)	2720 (53.6)	275 (27.9)	<0.001	2233 (49.8)	2055 (53.3)	178 (28.0)	<0.001
	Single	3063 (50.6)	2351 (46.4)	712 (72.1)		2255 (50.2)	1798 (46.7)	457 (72.0)	
Cohabitant status	With family	5055 (83.4)	4250 (83.8)	805 (81.6)	0.090	3648 (81.3)	3143 (81.6)	505 (79.5)	0.242
	Solitude	1003 (16.6)	821 (16.2)	182 (18.4)		840 (18.7)	710 (18.4)	130 (20.5)	
Health status and lifestyle									
BMI	Normal	3436(57.3)	2938(57.9)	531(53.8)	0.018	2506(55.8)	2173(56.4)	333(52.4)	0.069
	Abnormal	2589(42.7)	2133(42.1)	456(46.2)		1982(44.2	1680(43.6)	302(47.2)	
Medical expenses		300.00 (88.50, 1000.00)	300.00 (80.00, 1000.00)	300.00 (100.00, 1000.00)	0.303	500.00 (37.00, 2000.00)	500.00 (40.00, 2000.00)	400.00 (20.00, 2000.00)	0.299

Variables		Derivation set (n = 6058)				Temporal validation set (n = 4488)			
		Overall n=6058	Non-MCI n=5071	MCI n=987	*p*	Overall n=4488	Non-MCI n=3853	MCI n=635	*p*
Baseline CMMSE score		29.00 (27.00, 30.00)	29.00 (27.00, 30.00)	28.00 (26.00, 29.00)	<0.001	29.00 (27.00, 30.00)	29.00 (27.00, 30.00)	27.00 (26.00, 29.00)	<0.001
Sleep duration		8.00 (6.00, 9.00)	8.00 (6.00, 9.00)	8.00 (6.50, 10.00)	<0.001	8.00 (6.00, 9.00)	8.00 (6.00, 9.00)	8.00 (6.00, 10.00)	0.005
Chronic diseases	Yes	2348 (38.8)	1991 (39.3)	357 (36.2)	0.074	3401 (75.8)	2928 (76.0)	473 (74.5)	0.441
	No	3710 (61.2)	3080 (60.7)	630 (63.8)		1087 (24.2)	925 (24.0)	162 (25.5)	
BADL	Intact	5767 (95.2)	4883 (96.3)	884 (89.6)	<0.001	3954 (88.1)	3465 (89.9)	489 (77.0)	<0.001
	Impaired	291 (4.8)	188 (3.7)	103 (10.4)		534 (11.9)	388 (10.1)	146 (23.0)	
IADL	Intact	3453 (57.0)	3149 (62.1)	304 (30.8)	<0.001	2366 (52.7)	2203 (57.2)	163 (25.7)	<0.001
	Impaired	2605 (43.0)	1922 (37.9)	683 (69.2)		2122 (47.3)	1650 (42.8)	472 (74.3)	
PPT	Intact	4623 (76.3)	4037 (79.6)	586 (59.4)	<0.001	3307 (73.7)	2985 (77.5)	322 (50.7)	<0.001
	Impaired	1435 (23.7)	1034 (20.4)	401 (40.6)		1181 (26.3)	868 (22.5)	313 (49.3)	
Vision	Intact	4792 (79.1)	4132 (81.5)	660 (66.9)	<0.001	3373 (75.2)	2990 (77.6)	383 (60.3)	<0.001
	Impaired	1266 (20.9)	939 (18.5)	327 (33.1)		1115 (24.8)	863 (22.4)	252 (39.7)	
Hearing	Intact	5372 (88.7)	4613 (91.0)	759 (76.9)	<0.001	1461 (32.6)	1134 (29.4)	327 (51.5)	<0.001
	Impaired	686 (11.3)	458 (9.0)	228 (23.1)		3027 (67.4)	2719 (70.6)	308 (48.5)	
Masticatory function	Intact	3493 (57.7)	3135 (61.8)	358 (36.3)	<0.001	2825 (62.9)	2525 (65.5)	300 (47.2)	<0.001
	Impaired	2565 (42.3)	1936 (38.2)	629 (63.7)		1663 (37.1)	1328 (34.5)	335 (52.8)	
Eat fresh fruits	Usually	2603 (43.0)	2256 (44.5)	347 (35.2)	<0.001	1773 (39.5)	1569 (40.7)	204 (32.1)	<0.001
	Sometimes	2244 (37.0)	1825 (36.0)	419 (42.5)		1626 (36.2)	1374 (35.7)	252 (39.7)	
	Never	1211 (20.0)	990 (19.5)	221 (22.4)		1089 (24.3)	910 (23.6)	179 (28.2)	

Variables		Derivation set (n = 6058)				Temporal validation set (n = 4488)			
		Overall n=6058	Non-MCI n=5071	MCI n=987	*p*	Overall n=4488	Non-MCI n=3853	MCI n=635	*p*
Eat fresh vegetables	Usually	5609 (92.6)	4712 (92.9)	897 (90.9)	0.065	4128 (92.0)	3568 (92.6)	560 (88.2)	<0.001
	Sometimes	385 (6.4)	306 (6.0)	79 (8.0)		264 (5.9)	215 (5.6)	49 (7.7)	
	Never	64 (1.1)	53 (1.0)	11 (1.1)		96 (2.1)	70 (1.8)	26 (4.1)	
Eat animal protein	Usually	3415 (56.4)	2901 (57.2)	514 (52.1)	0.012	2559 (57.0)	2182 (56.6)	377 (59.4)	0.189
	Sometimes	2574 (42.5)	2113 (41.7)	461 (46.7)		1884 (42.0)	1635 (42.4)	249 (39.2)	
	Never	69 (1.1)	57 (1.1)	12 (1.2)		45 (1.0)	36 (0.9)	9 (1.4)	
Eat plant protein	Usually	1210 (20.0)	1027 (20.3)	183 (18.5)	0.365	701 (15.6)	607 (15.8)	94 (14.8)	0.085
	Sometimes	4380 (72.3)	3659 (72.2)	721 (73.0)		3363 (74.9)	2897 (75.2)	466 (73.4)	
	Never	468 (7.7)	385 (7.6)	83 (8.4)		424 (9.4)	349 (9.1)	75 (11.8)	
Drink tea	Usually	2200 (36.3)	1893 (37.3)	307 (31.1)	<0.001	1219 (27.2)	1098 (28.5)	121 (19.1)	<0.001
	Sometimes	933 (15.4)	799 (15.8)	134 (13.6)		747 (16.6)	645 (16.7)	102 (16.1)	
	Never	2925 (48.3)	2379 (46.9)	546 (55.3)		2522 (56.2)	2110 (54.8)	412 (64.9)	
House work	Usually	3631 (59.9)	3165 (62.4)	466 (47.2)	<0.001	2716 (60.5)	2424 (62.9)	292 (46.0)	<0.001
	Sometimes	959 (15.8)	794 (15.7)	165 (16.7)		563 (12.5)	482 (12.5)	81 (12.8)	
	Never	1468 (24.2)	1112 (21.9)	356 (36.1)		1209 (26.9)	947 (24.6)	262 (41.3)	
Field work	Usually	3244 (53.5)	2819 (55.6)	425 (43.1)	<0.001	2400 (53.5)	2144 (55.6)	256 (40.3)	<0.001
	Sometimes	1312 (21.7)	1079 (21.3)	233 (23.6)		806 (18.0)	678 (17.6)	128 (20.2)	
	Never	1502 (24.8)	1173 (23.1)	329 (33.3)		1282 (28.6)	1031 (26.8)	251 (39.5)	
Garden work	Usually	768 (12.7)	719 (14.2)	49 (5.0)	<0.001	859 (19.1)	786 (20.4)	73 (11.5)	<0.001
	Sometimes	406 (6.7)	370 (7.3)	36 (3.6)		314 (7.0)	280 (7.3)	34 (5.4)	
	Never	4884 (80.6)	3982 (78.5)	902 (91.4)		3315 (73.9)	2787 (72.3)	528 (83.1)	
Read newspapers or	Usually	834 (13.8)	786 (15.5)	48 (4.9)	<0.00	651 (14.5)	608 (15.8)	43 (6.8)	<0.00

Variables		Derivation set (n = 6058) Overall n=6058	Non-MCI n=5071	MCI n=987	*p*	Temporal validation set (n = 4488) Overall n=4488	Non-MCI n=3853	MCI n=635	*p*
books					1				1
	Sometimes	715 (11.8)	652 (12.9)	63 (6.4)		534 (11.9)	495 (12.8)	39 (6.1)	
	Never	4509 (74.4)	3633 (71.6)	876 (88.8)		3303 (73.6)	2750 (71.4)	553 (87.1)	
Raise domestic animals or pets	Usually	1584 (26.1)	1383 (27.3)	201 (20.4)	<0.001	1196 (26.6)	1070 (27.8)	126 (19.8)	<0.001
	Sometimes	485 (8.0)	404 (8.0)	81 (8.2)		287 (6.4)	257 (6.7)	30 (4.7)	
	Never	3989 (65.8)	3284 (64.8)	705 (71.4)		3005 (67.0)	2526 (65.6)	479 (75.4)	
Play cards or mahjong	Usually	517 (8.5)	464 (9.2)	53 (5.4)	<0.001	384 (8.6)	357 (9.3)	27 (4.3)	<0.001
	Sometimes	847 (14.0)	764 (15.1)	83 (8.4)		558 (12.4)	501 (13.0)	57 (9.0)	
	Never	4694 (77.5)	3843 (75.8)	851 (86.2)		3546 (79.0)	2995 (77.7)	551 (86.8)	
TV watching or radio listening	Usually	3936 (65.0)	3483 (68.7)	453 (45.9)	<0.001	3001 (66.9)	2728 (70.8)	273 (43.0)	<0.001
	Sometimes	969 (16.0)	680 (13.4)	289 (29.3)		711 (15.8)	565 (14.7)	146 (23.0)	
	Never	1153 (19.0)	908 (17.9)	245 (24.8)		776 (17.3)	560 (14.5)	216 (34.0)	
Social activities	Usually	234 (3.9)	221 (4.4)	13 (1.3)	<0.001	178 (4.0)	168 (4.4)	10 (1.6)	<0.001
	Sometimes	817 (13.5)	736 (14.5)	81 (8.2)		621 (13.8)	565 (14.7)	56 (8.8)	
	Never	5007 (82.7)	4114 (81.1)	893 (90.5)		3689 (82.2)	3120 (81.0)	569 (89.6)	
Smoke	Yes	1402 (23.1)	1256 (24.8)	146 (14.8)	<0.001	1000 (22.3)	905 (23.5)	95 (15.0)	<0.001
	No	4656 (76.9)	3815 (75.2)	841 (85.2)		3488 (77.7)	2948 (76.5)	540 (85.0)	
Alcohol use	Yes	1290 (21.3)	1137 (22.4)	153 (15.5)	<0.001	916 (20.4)	818 (21.2)	98 (15.4)	0.001
	No	4768 (78.7)	3934 (77.6)	834 (84.5)		3572 (79.6)	3035 (78.8)	537 (84.6)	
Exercise	Yes	2235 (36.9)	1983 (39.1)	252 (25.5)	<0.001	1791 (39.9)	1598 (41.5)	193 (30.4)	<0.001

Variables		Derivation set (n = 6058)				Temporal validation set (n = 4488)			
		Overall n=6058	Non-MCI n=5071	MCI n=987	*p*	Overall n=4488	Non-MCI n=3853	MCI n=635	*p*
	No	3823 (63.1)	3088 (60.9)	735 (74.5)		2697 (60.1)	2255 (58.5)	442 (69.6)	
Resilience score		20.00 (17.00, 22.00)	20.00 (18.00, 22.00)	19.00 (16.00, 21.00)	<0.001	20.00 (18.00, 21.00)	20.00 (18.00, 22.00)	18.00 (16.00, 21.00)	<0.001
Mental health									
Life satisfaction	Good	3660 (60.4)	3061 (60.4)	599 (60.7)	0.218	2742 (61.1)	2372 (61.6)	370 (58.3)	0.063
	So so	2052 (33.9)	1731 (34.1)	321 (32.5)		1525 (34.0)	1302 (33.8)	223 (35.1)	
	Bad	346 (5.7)	279 (5.5)	67 (6.8)		221 (4.9)	179 (4.6)	42 (6.6)	
Health satisfaction	Good	3360 (55.5)	2814 (55.5)	546 (55.3)	0.921	2162 (48.2)	1899 (49.3)	263 (41.4)	<0.001
	So so	1937 (32.0)	1617 (31.9)	320 (32.4)		1698 (37.8)	1445 (37.5)	253 (39.8)	
	Bad	761 (12.6)	640 (12.6)	121 (12.3)		628 (14.0)	509 (13.2)	119 (18.7)	
Sleep satisfaction	Good	4074 (67.2)	3423 (67.5)	651 (66.0)	0.123	2819 (62.8)	2445 (63.5)	374 (58.9)	0.087
	So so	1365 (22.5)	1120 (22.1)	245 (24.8)		1095 (24.4)	925 (24.0)	170 (26.8)	
	Bad	619 (10.2)	528 (10.4)	91 (9.2)		574 (12.8)	483 (12.5)	91 (14.3)	
Financial satisfaction	Rich	872 (14.4)	752 (14.8)	120 (12.2)	0.003	848 (18.9)	742 (19.3)	106 (16.7)	0.001
	So so	4241 (70.0)	3559 (70.2)	682 (69.1)		3040 (67.7)	2626 (68.2)	414 (65.2)	
	Poor	945 (15.6)	760 (15.0)	185 (18.7)		600 (13.4)	485 (12.6)	115 (18.1)	
Community and family support									
Child support		1300.00 (600.00, 2500.00)	1300.00 (600.00, 2500.00)	1500.00 (700.00, 2500.00)	0.004	1200.00 (300.00, 2700.00)	1200.00 (300.00, 2900.00)	1000.00 (275.00, 2300.00)	0.010
Community services	Yes	1744 (28.8)	1464 (28.9)	280 (28.4)	0.780	2188 (48.8)	1886 (48.9)	302 (47.6)	0.544
	No	4314 (71.2)	3607 (71.1)	707 (71.6)		2300 (51.2)	1967 (51.1)	333 (52.4)	

Abbreviations: MCI, Mild Cognitive Impairment; BMI, Body Mass Index; CMMSE, Chinese Mini-Mental State Examination; BADL, Basic Activity of Daily Living; IADL, Instrumental Activity of Daily Living; PPT, Physical Performance Test.

Table S3: Multivariate logistic regression analysis of variables associated.

variables		Coefficient	SE	Z value	OR	95% CI		*p*
						LL	UL	
Intercept		-7.474086	0.397799	-18.789	0.001	0.000	0.001	<0.001
Age		0.056940	0.004561	12.484	1.060	1.050	1.070	<0.001
Sex	Male	Ref	Ref	Ref	Ref	NA	NA	NA
	Female	0.260831	0.086661	3.010	1.300	1.100	1.540	0.003
Educational level	Illiteracy	Ref	Ref	Ref	Ref	NA	NA	NA
	Primary school	-0.454130	0.096681	-4.697	0.640	0.530	0.770	<0.001
	Middle school or above	-0.733703	0.165223	-4.441	0.480	0.350	0.660	<0.001
Exercise	Yes	Ref	Ref	Ref	Ref	NA	NA	NA
	No	0.190398	0.087359	2.179	1.210	1.020	1.440	0.029
Garden work	Usually	Ref	Ref	Ref	Ref	NA	NA	NA
	Sometimes	0.276074	0.238625	1.157	1.320	0.830	2.100	0.247
	Never	0.600872	0.161506	3.720	1.820	1.330	2.500	<0.001
TV watching or radio listening	Usually	Ref	Ref	Ref	Ref	NA	NA	NA
	Sometimes	0.243289	0.098455	2.471	1.280	1.050	1.550	0.013
	Never	0.254242	0.095256	2.669	1.290	1.070	1.550	0.008
IADL	Intact	Ref	Ref	Ref	Ref	NA	NA	NA
	Impaired	0.363276	0.089479	4.060	1.440	1.210	1.710	<0.001
Hearing	Intact	Ref	Ref	Ref	Ref	NA	NA	NA
	Impaired	0.318173	0.101488	3.135	1.370	1.130	1.680	0.002
Masticatory function	Intact	Ref	Ref	Ref	Ref	NA	NA	NA
	Impaired	0.394562	0.080298	4.914	1.480	1.270	1.740	<0.001

Abbreviations: SE, Standard Error; OR, Odds Ratio; CI, Confidence Interval; LL, Lower Limit; UL, Upper Limit; IADL, Instrumental Activity of Daily Living

Table S4: Points of CGMCI-Risk parameters.

**variables**	**Parameter**	**Points**
Age		1.818181818 * Trueage-109.090909091
Sex	Male	0
	Female	8.769365
Educational level	Illiteracy	25.84193
	Primary school	12.92096
	Middle school or above	0
Exercise	Yes	0
	No	6.292808
Garden work	Usually	0
	Sometimes	9.643149
	Never	19.286299
TV watching or radio listening	Usually	0
	Sometimes	4.250002
	Never	8.500005
IADL	Intact	0
	Impaired	11.78163
Hearing	Intact	0
	Impaired	10.3652
Masticatory function	Intact	0
	Impaired	12.65261

Abbreviations: IADL, Instrumental Activity of Daily Living.
